# Multi-sensor datasets of ultrasonic and mmWave reflected signals for material classification

**DOI:** 10.1016/j.dib.2026.112760

**Published:** 2026-04-08

**Authors:** Mohammed H. Sadiq, Shaheen A. Abdulkareem, Ahmad B. Al-Khalil

**Affiliations:** Department of Computer Science, University of Duhok, Duhok 42001, Iraq

**Keywords:** Contactless sensing, Sensor data, Thickness profiling, Echo analysis, Signal processing

## Abstract

This article details a publicly accessible dataset of reflected signals from ultrasonic and millimetre-wave (mmWave) sensors used for material classification. The dataset was gathered using two sensing methods: the URM09 ultrasonic sensor operating at 40 kHz and the DFRobot mmWave C4001 radar sensor operating at 24 GHz. Six common materials were chosen for data collection: wood, plastic, metal, glass, cardboard, and asbestos. Each material was tested at three thicknesses and one size (25 cm × 25 cm). All measurements were taken from a fixed distance of 55 cm in a controlled indoor setting with foam-lined enclosures to reduce external noise. The raw analogue signals were processed with Savitzky-Golay filtering and sliding window techniques. Time-domain features (mean, standard deviation, energy, RMS, kurtosis, skewness) and frequency-domain features (spectral centroid, spectral bandwidth, dominant frequency) were extracted from each sample. For URM09 the dataset includes 1789 refined data points across all six material-thickness combinations and 565 records for six material-only classification. On the other hand, For C4001 the dataset includes 1504 refined data points across all five material-thickness combinations and 470 records for five material-only classification. This dataset supports research in robotics, industrial inspection, non-destructive testing, and sensor fusion applications where contactless material identification is essential.

Specifications Table

**Table:** 

Subject	Computer Sciences
Specific subject area	Material recognition using ultrasonic and mmWave sensors
Type of data	CSV files of processed signals (Tables, Raw, Analyzed, Filtered, Processed)
Data collection	The data collected used the URM09 ultrasonic sensor (DFRobot Gravity Series) which emits 40 kHz sound waves and measures the analogue of reflected echoes. Additionally, The mmWave C4001 sensor (DFRobot) emits 24 GHz electromagnetic waves and measures reflections. An ESP32 microcontroller handled sensor interfacing and wireless data transmission via TCP/IP socket.
Data source location	Department of Computer Science, University of Duhok, Duhok, Kurdistan Region, Iraq
Data accessibility	Data identification number: https://doi.org/10.5281/zenodo.19209067 Direct URL to data: https://zenodo.org/records/19209067
Related research article	The article related to this paper is just submitted to a journal and the status is still submitted to journal. I have already uploaded a copy of the related article as pdf on the system.

## 1 Value of the Data

• Material identification using contactless sensing is crucial in robotics, industrial automation, non-destructive testing, and smart environmental monitoring. Ultrasonic and mmWave sensors offer precise, dependable measurements across various ambient conditions, including low visibility and optical blockages. These sensors support applications such as obstacle detection, object classification, quality assurance, and robot navigation.
• No existing public dataset combines both ultrasonic (URM09) and mmWave (C4001) sensor data across multiple materials with different thicknesses. This dataset addresses that gap by providing signal data from both sensor types under controlled conditions. Researchers can use this dataset to develop classification algorithms, compare sensor modalities, explore sensor-fusion techniques, and benchmark machine learning models for material recognition.
• The dataset reveals complementary strengths between the two sensor types. The URM09 ultrasonic sensor performs best with dense, rigid materials such as wood (100%), metal (99%), and plastic (96%) for thickness classification. However, it struggles with porous materials like cardboard (68%) and asbestos (64%). Conversely, the mmWave C4001 sensor excels with porous and permeable materials such as cardboard (100%) and plastic (97%), but struggles with highly reflective materials such as metal (79%). Users interested in robust material classification across diverse material types may benefit from sensor-fusion approaches that combine modalities.
• Parallel data from two distinct sensing modalities enable the development and benchmarking of multimodal fusion algorithms. Researchers can use this dataset as a source domain for transfer learning experiments targeting different materials, sensors, or environmental conditions. Processed signal data can serve as a testbed for comparing different filtering techniques, feature extraction methods, and dimensionality reduction approaches.
• The dataset can be used to train models that detect unknown or defective materials based on deviations from learned signal patterns. Although this dataset focuses on material classification, the underlying signal characteristics may be helpful in general time-series analysis research. The dataset serves as a practical resource for teaching signal processing, feature extraction, and machine learning classification.

## 2 Background

Material identification using contactless sensing is crucial in robotics, industrial automation, non-destructive testing, and smart environmental monitoring. Ultrasonic and mmWave sensors offer precise, dependable measurements across various ambient conditions, including low visibility and optical blockages. These sensors support applications such as obstacle detection, object classification, quality assurance, and robot navigation.

Previous research has examined material classification using ultrasonic and microwave sensors independently. Sahoo and Udgata (2024) employed ultrasonic echo signals with a 1D CNN to classify five materials (glass, wood, metal, sponge, and fabric), achieving 96% accuracy [1]. Zhu et al. (2023) employed Empirical Mode Decomposition with SVM to classify wood, paper, and foam, reaching 97.3% accuracy [2]. For mmWave sensing, Erdélyi et al. [3] developed a material-reflection-feature approach using a 60 GHz radar, achieving 92.87% accuracy across five materials [3]. Arab et al. (2021) used 24 GHz radar with SVM for object classification based on radar cross-sections [4].

Nevertheless, no existing public dataset combines both ultrasonic (URM09) and mmWave (C4001) sensor data across multiple materials with different thicknesses. This dataset addresses that gap by providing signal data from both sensor types under controlled conditions. Researchers can use this dataset to develop classification algorithms, compare sensor modalities, explore sensor-fusion techniques, and benchmark machine learning models for material recognition.

## 3 Data Description

The dataset is stored in CSV (Comma-Separated Values) format for easy accessibility and compatibility with common data analysis tools. The data is organised into separate files based on sensor type and experimental configuration. Table 1 provides an overview of all dataset files included in this repository.

**Table 1:** Dataset files overview.

File Name	Sensor	Description	Records	Materials
URM09_AllMaterials_AllThickness.csv	URM09	All materials with all thickness level	1789	6
URM09_Materials_Only.csv	URM09	Materials regardless of thickness	565	6
C4001_AllMaterials_AllThickness.csv	C4001	All materials with all thickness level	1504	5
C4001_Materials_Only.csv	C4001	Materials regardless of thickness	470	5
Raw Data	URM09	Raw Data of All six materials all Thickness Separately	979 per each class	6
Raw Data	C4001	Raw Data of All five materials all Thickness Separately	979 per each class	5

The repository also encompasses the raw time-series signal files for all materials and thickness levels. For each sensor, 979 raw analogue signal recordings were acquired per material class and are provided as individual CSV files, systematically organized by sensor type, material, and thickness combination. Each raw file comprises solely two columns: the signal amplitude values and the corresponding material label. These raw files are separate from the processed feature tables and enable users to implement alternative filtering techniques, feature extraction strategies, or end-to-end deep learning pipelines directly on the unprocessed signals data.

Each CSV file contains the following columns as described in Table 2, while raw data of each material (thickness) has only signal and label columns:

**Table 2:** Column description.

Column Name	Data Type	Description
Mean	Float	Average signal amplitude
StdDev	Float	Standard deviation
Max	Float	Maximum signal value
Min	Float	Minimum signal value
Energy	Float	Total signal energy
Median	Float	Median signal value
RMS	Float	Root Mean Square value
ZCR	Float	Zero-Crossing Rate
Skewness	Float	Skewness of amplitude distribution
Kurtosis	Float	Kurtosis of amplitude distribution
DominantFreq	Float	Dominant frequency component
AvgAmplitude	Float	Average amplitude in frequency domain
SpectralCentroid	Float	Center of mass of frequency domain
SpectralBandwidth	Float	Spread of frequency spectrum
Label	String	Material Type (Wood, Plastic, Metal, Glass, Cardboard, Asbestos)

Each row represents a processed sample derived from 10 consecutive raw signal readings using a sliding window. The raw signals were first filtered with a Savitzky-Golay filter, and the mean within each window was then calculated to produce the refined feature vectors.

The dataset files are independent and self-contained. The "AllMaterials_AllThickness" files include the complete dataset with material and thickness labels. The "Materials_Only" files consist of subsets with one thickness level per material for pure material classification tasks. Users can select the appropriate file based on their research needs: thickness classification requires the entire dataset, while material classification can use either file.

Six materials were selected: wood, plastic, metal, glass, cardboard, and asbestos. Each material was prepared in three Thickness levels and one size (25 cm × 25 cm). The thickness values are as follows: Metal (0.04 cm, 0.05 cm, 0.1 cm), Wood (0.4 cm, 0.6 cm, 1.7 cm), Glass (0.3 cm, 0.5 cm, 0.9 cm), Plastic (0.7 cm, 1.8 cm, 2.5 cm), Cardboard (0.2 cm, 0.3 cm, 0.5 cm), and Asbestos (0.1 cm, 0.2 cm, 0.3 cm).

### 3.1 Data quality and validation

Table 3 shows the classification performance for material identification regardless of thickness.

**Table 3:** Material classification accuracy.

Sensor	Materials	Accuracy	Precision	Recall
URM09	6 materials	100%	100%	100%
C4001	5 materials	99%	99%	99%

The high classification accuracy observed across both sensors indicates that the extracted features provide strong inter-class separability for material identification, confirming the discriminative quality of the dataset.

Table 4 shows classification performance when distinguishing materials across all thickness levels simultaneously.

**Table 4:** Material and thickness classification accuracy.

Sensor	Materials	Accuracy	Precision	Recall
URM09	6 materials, 3 thicknesses	72%	72%	71%
C4001	5 materials, 3 thicknesses	77%	77%	75%

The moderate accuracy observed in the joint material-and-thickness classification task reflects the increased complexity of discriminating 18 simultaneous classes, and is consistent with the expected signal overlap between closely spaced thickness levels. These results indicate that the dataset captures genuine physical variation in reflected signals, providing a realistic and challenging benchmark for multi-class classification research.

Table 5 presents thickness classification accuracy for individual materials using the URM09 sensor.

**Table 5:** Thickness classification per material (URM09).

Materials	Accuracy	Precision	Recall	F1-Score
Wood	100%	100%	100%	100%
Metal	99%	99%	99%	99%
Plastic	96%	96%	96%	96%
Glass	72%	73%	72%	72%
Cardboard	68%	68%	68%	68%
Asbestos	64%	64%	64%	64%

The variation in per-material thickness classification accuracy reveals material-dependent differences in ultrasonic signal discriminability. Dense, rigid materials such as wood and metal produce highly separable reflection signatures across thickness levels, while porous materials such as cardboard and asbestos generate more overlapping responses. This variability is an inherent property of the materials themselves and reflects the true discriminative structure of the dataset, making it a useful resource for studying sensor sensitivity across diverse material compositions.

Table 6 presents thickness classification accuracy for individual materials using the C4001 sensor.

**Table 6:** Thickness classification per material (C4001).

Materials	Accuracy	Precision	Recall	F1-Score
Cardboard	100%	100%	100%	100%
Plastic	97%	97%	97%	97%
Wood	85%	85%	85%	85%
Glass	84%	85%	84%	84%
Metal	79%	78%	79%	78%

**Fig. 1:**
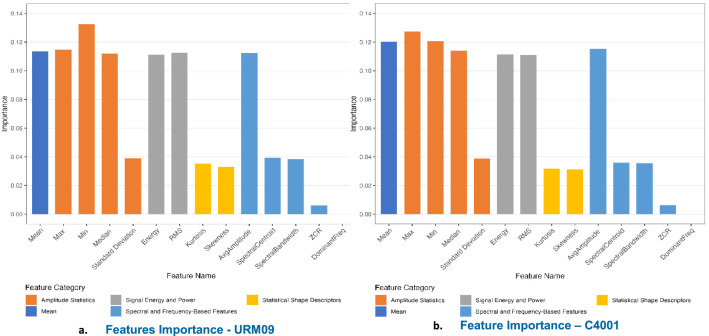
Feature importance scores for the extracted time-domain and frequency-domain features. These values were derived from the Random Forest classifier used in the validation section to assess the discriminative quality of the dataset.".

The mmWave sensor demonstrates complementary discriminability characteristics relative to the ultrasonic sensor, with stronger thickness separability for porous materials such as cardboard and plastic, and comparatively lower separability for highly reflective surfaces such as metal. This complementary pattern across sensor modalities reinforces the value of the dataset for sensor fusion research, where the contrasting signal behaviours of both sensors can be exploited to improve overall classification robustness Fig. 1.

## 4 Experimental Design, Materials and Methods

### 4.1 Hardware setup

**Fig. 2:**
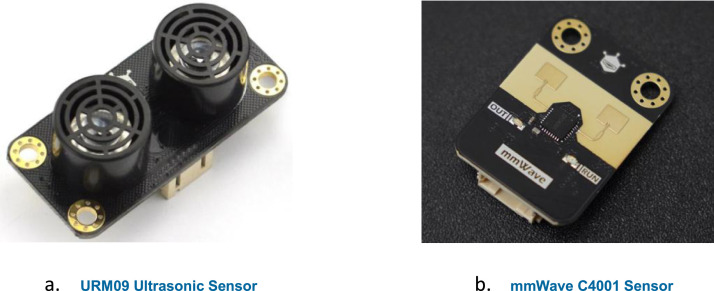
Sensors in use.

The data acquisition system consists of three main components: two sensors and one microcontroller. The URM09 ultrasonic sensor (DFRobot Gravity Series) operates at 40 kHz, with a detection range of 2 cm to 500 cm and ±1% accuracy (Fig. 2a.) [5]. It provides 2–500 cm range with ±1% accuracy, 30–50 Hz sampling rate, and integrated temperature compensation. The sensor operates at 3.3–5.5 V with <20 mA current consumption, featuring 30° vertical and 60° horizontal detection angles.

The mmWave C4001 sensor (DFRobot) operates at 24 GHz and has a detection range of up to 25 m (Fig. 2b.) [6]. It achieves 25 m detection range with 40° vertical and 100° horizontal coverage, operating at 3.3/5 V. It provides continuous analogue output with good noise immunity.

**Fig. 3:**
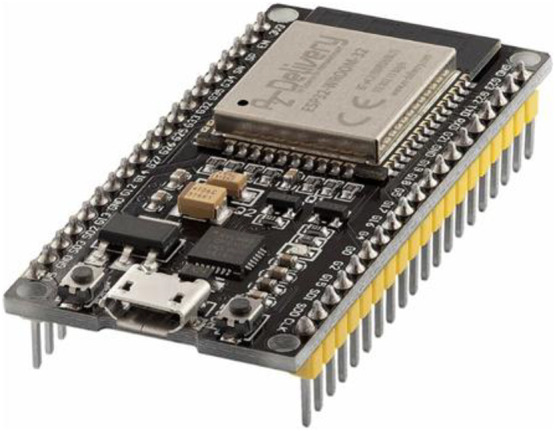
ESP32 microcontroller.

Both sensors were connected to an ESP32 microcontroller, which features a dual-core Xtensa LX6 processor, 240 MHz, 520 KB SRAM, and a 12-bit analogue-to-digital converter (ADC). The ESP32 operated as a Wi-Fi hotspot, streaming sensor data to a laptop via a TCP/IP socket in real time, eliminating serial connection limitations (Fig. 3) [7].

### 4.2 Environment configuration

**Fig. 4:**
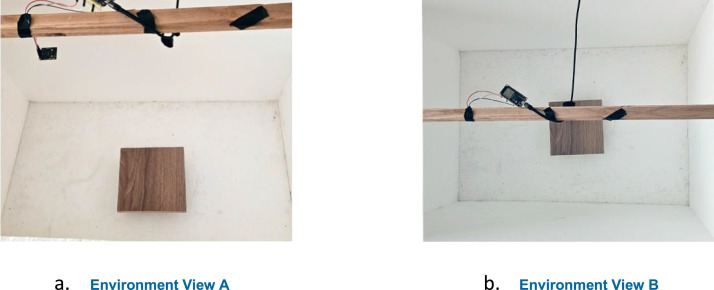
The controlled laboratory environment.

All experiments were conducted in a controlled laboratory setup designed to ensure reliable reflection measurement (Fig. 4). Both sensors possess a wide field-of-view, which can introduce unwanted multipath and off-axis reflections from surrounding objects. The sensors were placed inside a foam-lined enclosure to limit the field of view and reduce signal interference from surrounding objects [8]. To isolate the true material response, the sensors were positioned 55 cm from the target samples inside a foam-lined enclosure. This distance lies within the effective operating range of both sensing modalities and allows stable capture of reflected signals while preserving realistic propagation behaviour. The foam lining was used to absorb stray reflections and suppress multipath components, enabling the recorded signals to primarily represent direct sensor–material interactions.

### 4.3 Materials and samples

Six materials were selected: wood, plastic, metal, glass, cardboard, and asbestos. Each material was prepared in three thickness levels and one size (Table 7).

**Table 7:** Materials type, size, and thickness.

Item	Size	Thickness	Image
Metal	25 cm x 25 cm	Metal 1 - 0.04 cm Metal 2 - 0.05 cm Metal 3 - 0.1 cm	
Wood	25 cm x 25 cm	Wood 1 - 0.4 cm Wood 2 - 0.6 cm Wood 3 - 1.7 cm	
Glass	25 cm x 25 cm	Glass 1 - 0.3 cm Glass 2 - 0.5 cm Glass 3 - 0.9 cm	
Plastic	25 cm x 25 cm	Plastic 1 - 0.7 cm Plastic 2 - 1.8 cm Plastic 3 - 2.5 cm	
Cardboard	25 cm x 25 cm	Cardboard 1 - 0.2 cm Cardboard 2 - 0.3 cm Cardboard 3 - 0.5 cm	
Asbestos	25 cm x 25 cm	Asbestos 1 - 0.1 cm Asbestos 2 - 0.2 cm Asbestos 3 - 0.3 cm	

### 4.4 Data collection and procedure

Each material was tested individually to prevent interference. Before each recording session, the material type was recorded in the terminal. The sensors continuously captured analogue signals, and multiple sessions were carried out for each material, thickness, and size combination. For each sensor, 979 raw signal samples were recorded per material class.

The data collection was intentionally conducted under a controlled laboratory setup with a fixed sensor–target distance of 55 cm, perpendicular alignment, and a noise-attenuated enclosure. This design choice was motivated by the primary objective of the study: to examine whether low-cost microwave and mmWave sensors—originally engineered for simple motion and presence detection—can capture reflected signal characteristics with sufficient granularity to enable material classification. Specifically, the framework evaluates the sensors' ability to discriminate not only between diverse material compositions (types) but also between varying physical thicknesses, thereby testing the limits of low-cost hardware in complex industrial sensing tasks. The controlled environment minimizes the influence of external noise, multipath reflections, and background clutter, thereby isolating the contribution of material-dependent reflection properties. This consideration is particularly important given the wide field of view (FoV) of the employed sensors, which makes them highly sensitive to unintended reflections from surrounding objects. By constraining the sensing geometry, the experimental setup ensures that the recorded signals predominantly originate from the target material. The findings further highlight a practical design implication: for material-sensitive sensing applications, sensors with narrower or configurable FoV characteristics may offer improved selectivity and robustness. While the present dataset does not attempt to capture the full variability of real-world robotics or industrial inspection scenarios, it provides a foundational benchmark for understanding material reflection behaviour under controlled conditions and can serve as a reference point for future studies incorporating increased environmental and geometric variability.

### 4.5 Signal processing

To ensure reproducibility of the feature extraction pipeline, all signal processing parameters are explicitly defined. Raw reflected signals were first smoothed using a Savitzky–Golay filter with a window length of 11 samples and a polynomial order of 3 [9]. This configuration was selected to suppress high-frequency noise while preserving both linear trends and mild curvature in the reflected signal, which are characteristic of material-dependent responses. Following smoothing, a fixed-length sliding window of 10 consecutive samples was applied to the uniformly sampled signal. Non-overlapping windows were used to avoid redundancy between adjacent feature vectors, and the mean value within each window was used as the representative amplitude prior to feature extraction. From each windowed segment, a set of time-domain and frequency-domain features was computed as listed in Table 8.

**Table 8:** Signal processing parameters.

Stage	Parameter	Value	Rationale
Pre-processing	Smoothing filter	Savitzky–Golay	Preserves signal shape while reducing noise
Savitzky–Golay	Window length	11 samples	Odd-length window required; smooths noise without distorting peaks
Savitzky–Golay	Polynomial order	3	Captures both linear trends and mild curvature in reflected signals
Windowing	Sliding window size	10 samples	Balances temporal resolution and noise reduction
Windowing	Window stride	10 samples (non-overlapping)	Prevents redundancy between adjacent feature vectors
Windowing	Aggregation	Mean value	Represents average reflected signal strength per window
Feature domain	Time-domain features	Mean, StdDev, Min, Max, Median, RMS, Energy, ZCR, Skewness, Kurtosis	Captures amplitude and distribution characteristics
Feature domain	Frequency-domain features	Dominant Frequency, Avg Amplitude, Spectral Centroid, Spectral Bandwidth	Captures spectral characteristics of reflected signals

### 4.6 Feature extraction

Features were extracted from both time and frequency domains. Time-domain features include: mean, maximum, minimum, median, standard deviation, energy, RMS, zero-crossing rate, skewness, and kurtosis. Frequency-domain features were obtained using the Fast Fourier Transform (FFT) and include: average amplitude, dominant frequency, spectral centroid, and spectral bandwidth (Table 9) [10].

**Table 9:** Feature extraction.

Domain	Features	Count
Time-domain	Mean Amplitude, Standard Deviation, Peak-to-Peak Amplitude (Max and Min), Signal Energy, Median, RMS, Zero-Crossing Rate, Skewness, Kurtosis	10
Frequency-domain	Average Amplitude, Dominant Frequency, Spectral Centroid, Spectral Bandwidth	4
Total Features per Sensor		14

### 4.7 Sensor specifications and accuracy

The URM09 ultrasonic sensor has a measurement resolution of 1 cm and an accuracy of ±1% within its 2 cm to 500 cm detection range. The sensor features integrated temperature compensation for stable operation under varying conditions. The mmWave C4001 sensor operates at 24 GHz, with a horizontal detection range of 100 degrees and a vertical range of 40 degrees. Both sensors were sampled using the ESP32′s 12-bit ADC, providing sufficient resolution to detect signal variations.

### 4.8 Data quality control

Several measures were introduced to guarantee data quality. Firstly, all experiments took place within a foam-lined enclosure to reduce multipath effects and external reflections. Secondly, a fixed distance of 55 cm and perpendicular alignment were maintained throughout all trials. Thirdly, materials remained stationary during data collection to prevent motion-related noise. Lastly, multiple recording sessions were conducted to ensure reproducibility.

### 4.9 Classification validation

To evaluate the discriminative capability of the dataset, a Random Forest classifier [11] combined with stratified 10-fold cross-validation [12] was employed as a baseline validation method. The objective of this experiment is not to optimize classification performance but to confirm that the extracted features possess adequate signal separability to facilitate material identification tasks. Standard hyperparameters were utilized, without significant parameter tuning.

For model evaluation, stratified 10-fold cross-validation was employed. The dataset was first transformed into a feature matrix, where each row corresponds to a feature vector extracted from a fixed-length sliding window applied to the uniformly sampled reflected signal, and each row is associated with a single material label. The data were then partitioned into ten folds at the feature-vector level, ensuring that each fold preserves the class distribution of the full dataset. In each iteration, nine folds were used for training and the remaining fold for testing, and the reported performance metrics represent the average values across all folds. To minimise bias, feature extraction was performed prior to cross-validation, and no information from the test folds was used during model training. The high classification accuracy observed for the ultrasonic sensor, including the 100% accuracy reported for the six-class material classification task, should be interpreted in the context of the dataset construction procedure. An initial set of experiments was conducted using all materials across multiple thicknesses, including joint material-and-thickness classification and per-material thickness discrimination. These experiments exhibited varying performance across materials and thickness levels, as reflected in the corresponding confusion matrices. Based on this analysis, the thickness level that yielded the most reliable and discriminative response for each material was selected, and a reduced dataset composed of these best-performing material–thickness combinations was subsequently constructed for focused material classification. This selection process was guided by empirical separability rather than model optimisation. Each classification sample corresponds to a feature vector derived from a fixed-length sliding window applied to uniformly sampled reflected signals, and no raw measurements or feature vectors are duplicated across training and test folds. Importantly, when more challenging classification settings are considered—such as joint material-and-thickness classification—the performance decreases substantially, indicating that the reported high accuracy is not the result of data leakage or overly optimistic validation, but rather reflects favourable signal separability under controlled experimental conditions.

### 4.10 Feature importance

Feature importance analysis was conducted to identify the most discriminative features. For both sensors, time-domain features (mean, max, min, energy, RMS) and frequency-domain features (average amplitude) contributed most significantly to classification performance. These results confirm that both time-domain and frequency-domain features carry meaningful discriminative information, supporting the utility of the dataset for a range of classification approaches.

### 4.11 Data collection code

Both the data acquisition firmware (ESP32/Arduino) and the signal-processing and machine-learning pipeline (Python) are hosted in a single GitHub repository.

The firmware for data collection was developed on the ESP32 microcontroller using the Arduino IDE, where the ESP32 operated as a Wi-Fi access point and transmitted real-time sensor measurements via TCP/IP socket to a laptop for recording. The data preprocessing, feature extraction, and machine-learning classification pipeline were implemented in Python.

Full source code (firmware + processing + classification):

https://zenodo.org/records/19209067

### 4.12 Third-party software and libraries

Table 10 lists all third-party software and libraries used in this study.

**Table 10:** Software and library versions.

Software/Library	Version	Purpose
Arduino IDE	2.3	ESP32 firmware development
Python	3.11	Data processing and analysis
Numpy	1.26	Numerical computations
Scipy	1.11	Savitzky-Golay filter implementation
Pandas	2.1	Data manipulation and CSV handling
Scikit-learnin	1.3	Random Forest classifier, cross-validation, grid search
Matplotlib	3.8	Data visualization

### 4.13 Key functions used

The following key functions were used in the processing pipeline:

• scipy.signal.savgol_filter(): Savitzky-Golay smoothing filter.
• numpy.fft.fft(): Fast Fourier Transform for frequency domain features.
• sklearn.ensemble.RandomForestClassifier(): Random Forest classification.
• sklearn.model_selection.cross_val_score(): 10-fold cross-validation.
• sklearn.model_selection.GridSearchCV(): Hyperparameter optimisation.

### 4.14 Repository structure

The code repository contains the following structure:

├—— Dataset&Sourcecode
  │ └—— Datasets
  │ └—— Source Code
  └—— README.md

## Limitations

First, the mmWave C4001 sensor could not interact with asbestos material. Therefore, asbestos data is only available for the URM09 ultrasonic sensor. Researchers working on sensor fusion should account for this missing class in the C4001 dataset.

Second, all data were collected at a fixed distance of 55 cm in a controlled indoor environment with foam-lined enclosures. The signal characteristics may differ in open environments, longer distances, or varying ambient conditions. Users applying this data to real-world applications should consider additional calibration or domain adaptation techniques.

Third, the sensors have wide fields of view (URM09: 60° horizontal, 30° vertical; C4001: 100° horizontal, 40° vertical). The foam enclosure effectively limited unwanted reflections during data collection. In uncontrolled settings, multipath effects and background interference may affect classification performance.

Fourth, the ESP32 microcontroller uses a 12-bit ADC, which provides sufficient resolution for this application but may introduce quantisation effects for applications requiring higher precision.

## Research Applications and Use Cases

• **Robotics researchers** who require reliable contactless material identification capabilities for autonomous systems and navigation tasks.
• **Industrial inspection and quality control engineers** who need non-contact sensing solutions for automated material verification in manufacturing environments.
• **Non-destructive testing (NDT) specialists** seeking multi-sensor datasets to develop and validate new inspection methodologies without physical contact with materials.
• **Sensor fusion researchers** interested in combining ultrasonic and mmWave sensing modalities to improve classification robustness and accuracy.
• **Machine learning and deep learning researchers** looking for well-structured, publicly available multi-modal benchmark datasets for developing and evaluating new classification algorithms.

## Ethics Statement

The authors have read and followed the ethical requirements for publication in Data in Brief. This research did not involve human subjects, animal experiments, or the collection of social media data. All materials used in this dataset were purchased or fabricated specifically for research purposes.

## CRediT Author Statement

**Mohammed H. Sadiq:** Methodology, Investigation, Data curation, Formal analysis, Validation, Writing – Original draft preparation, Writing – Reviewing and Editing. **Shaheen A. Abdulkareem:** Supervision, Validation, Writing – Original draft preparation, Writing – Reviewing and Editing. **Ahmad B. Al-Khalil:** Supervision, Validation, Writing – Original draft preparation, Writing – Reviewing and Editing.

## Data Availability

ZenodoMulti-Sensor Dataset of Ultrasonic and mmWave for Material Classification (MatSense2025) (Original data).
